# Fasting Induces IL-1 Resistance and Free-Fatty Acid-Mediated Up-Regulation of IL-1R2 and IL-1RA

**DOI:** 10.3389/fimmu.2014.00315

**Published:** 2014-07-09

**Authors:** Jennifer J. Joesting, Morgan L. Moon, Stephen J. Gainey, Brittany L. Tisza, Neil A. Blevins, Gregory G. Freund

**Affiliations:** ^1^Department of Animal Sciences, University of Illinois at Urbana – Champaign, Urbana, IL, USA; ^2^Program in Integrative Immunology and Behavior, Department of Pathology, University of Illinois at Urbana – Champaign, Urbana, IL, USA; ^3^Division of Nutritional Sciences, University of Illinois at Urbana – Champaign, Urbana, IL, USA

**Keywords:** fasting, starvation, IL-1, IL-1 receptor type II, IL-1R2, free-fatty acids, non-esterified fatty acids

## Abstract

**Objective:** Weight-loss is a near societal obsession and many diet programs use significant calorie restriction including fasting/short term starvation to generate rapid effects. Fasting is also a well-recognized cause of immunosuppression especially within the innate immune system. In this study, we sought to determine if the IL-1 arm of the neuroimmune system was down-regulated by a 24 h fast and how fasting might generate this effect.

**Design:** Mice were allowed *ad libitum* access to food or had food withheld for 24 h. Expression of the endogenous IL-1 antagonists, IL-1 receptor type 2 (IL-1R2), and IL-1 receptor antagonist (IL-1RA) was determined as were sickness behaviors before and after IL-1β administration.

**Results:** Fasting markedly increased gene expression of IL-1R2 (83-fold in adipose tissue, 9.5-fold in liver) and IL-1RA (68-fold in liver). Fasted mice were protected from IL-1β-induced weight-loss, hypoglycemia, loss of locomotor, and social anxiety. These protections were coupled to a large positive interaction of fasting and IL-1β on IL-1R2 gene expression in adipose tissue and liver (2.6- and 1.6-fold, respectively). Fasting not only increased IL-1RA and IL-1R2 protein 2.5- and 3.2-fold, respectively, in liver but also increased IL-1R2 1.8-fold in adipose tissue. Fasting, in turn, triggered a 2.4-fold increase in plasma free-fatty acids (FFAs) and a 2.1-fold increase in plasma corticosterone. Inhibition, of glucocorticoid action with mifepristone did not impact fasting-dependent IL-1R2 or IL-1RA gene expression. Administration of the FFA, palmitate, to mice increased liver IL-1R2 and IL-1RA gene expression by 14- and 11-fold, respectively.

**Conclusion:** These findings indicate that fasting augments expression of endogenous IL-1 antagonists inducing IL-1 resistance. Fasting-induced increases in plasma FFAs appears to be a signal that drives immunosuppression during fasting/short term starvation.

## Introduction

Within the last 20 years, the zeitgeist of body weight has become a physiological metric that has permeated the culturally psyche of nearly all industrialized nations. Therefore, weight-loss has become a near societal obsession (1). Many popular diet programs initially use significant calorie restriction (CR) to facilitate relatively rapid weight-loss (2) including the recent trend of detox and/or cleansing diets, which may incorporate water only fasting for up to 1 week. In a broad context, however, fasting is a critical element of many religious and cultural practices (3, 4) and is recommended as therapy for a variety of ailments by practitioners of alternative and complementary medicine (5–7). Clinically, dietary regimens that incorporate intermittent fasting (IF) have gained significant popular attention with human studies showing a potential to reduce heart disease and diabetes (8) and to improve mood (7). Since fasting regimens appear to benefit cognitive function in aging mice (9) and in the 3xTgAD mouse model of Alzheimer’s disease (10), interest in CR and IF as a way to ward off human cognitive aging has also emerged (11).

While overeating and obesity are associated with a variety of maladies including type 2 diabetes (T2D), heart disease, stroke (12), cancer (13), Alzheimer’s disease (14), anxiety/mood disorders (15), and cognitive impairment (11), how overnutrition contributes is not entirely clear. Emerging evidence indicates that oxidative and metabolic stress in conjunction with dyslipidemia and excess glucose stimulates the unfolded protein response (16) and activates the NLRP3 inflammasome (17). Thus, IL-1-mediated inflammation is a key consequence of overnutrition, whether indirectly provoked by cellular injury (16) or directly triggered by excess nutrients acting as danger signals (18). Overnutrition-associated IL-1 is implicated in pancreatic beta cell loss in T2D, atherogenesis in heart disease and stroke, and neurodegeneration in Alzheimer’s disease but, in general, IL-1 is a critical effector of sickness symptoms that include loss of appetite, locomotion, and social/environmental engagement (19). Thus, ameliorations that inhibit IL-1 bioaction may positively impact health in those with a variety of aliments including neuropsychiatric disease (20, 21).

We recently demonstrated that mice fasted for 24 h have reduced gene expression of brain IL-1α (22), indicating that acute caloric restriction may be a physiological way to negatively regulate IL-1 bioaction. Although starvation appears detrimental to immunity, as evidenced by lipopolysaccharide (LPS)-induced death in bees and mice (23, 24), fasting appears to mitigate LPS-induced sickness symptoms in rats (25). In addition, fasting offers protection from ischemic (26) and hypoxic injuries (27), which we and others have shown are mediated in large part by IL-1 (28). Therefore, we examined if the physical and perceived salutary effects of fasting are due to IL-1 counter-regulation in both the systemic and neuroimmune systems.

## Materials and Methods

### Materials

All reagents and chemicals were purchased from Sigma-Aldrich (St Louis, MO, USA) except as noted. All primers were purchased from Applied Biosystems (Foster City, CA, USA).

### Animals

Procedures were conducted on protocols approved by the University of Illinois Institutional Animal Care and Use Committee. IL-1 receptor 1 knock out (KO), toll-like receptor (TLR-4) KO, IL-4 KO, and C57BL/6J [wild type (WT)] mice were purchased from Jackson Laboratory (Bar Harbor, ME, USA) and bred in-house. All KO mice were on a C57BL/6 background. Mice were group housed (×8 cage) in standard shoebox cages (length 46.9 cm; width 25.4 cm; height 12.5 cm) and allowed water and Harlan chow (Indianapolis, IN, USA) *ad libitum* except where otherwise noted. Housing temperature (72°F) and humidity (45–55%) were controlled as was a 12/12 h reversed dark–light cycle (light = 2200–1000 h). After shipping, mice were allowed at least 1 week to adjust to the above conditions prior to experimental pre-conditioning. Mice were handled/scruffed for 1 week prior to behavioral testing and biomarker determinations. Animals were sacrificed using CO_2_ except for those in which corticosterone was measured, which were sacrificed using ketamine/xylazine. Different cohorts of mice were used for behavioral testing and biomarker studies. The total number of mice utilized was 430.

### Fasting and mouse weight

As we have described (22), 2 days prior to fasting, mice were singly housed. Fasting was initiated by placing mice in a new cage without food but with *ad libitum* water. Mice were fasted for 24 h starting at 09:00 h. Immediately after the 24 h fasting, behavioral testing, biomarker studies, and/or injection studies were performed. Consequently, all studies occurred during the dark (active) cycle. Mouse weight was recorded immediately before and immediately after fasting using an Ohaus Adventurer Pro digital scale (Parsippany, NJ, USA).

### Injectables

Millipore recombinant mouse IL-1β (Billerica, MA, USA) in 100 μl of sterile Cellgro PBS (Manassas, VA, USA) (vehicle) was administered intraperitoneally (IP) at a dose of 0.9 μg/mouse immediately after fasting. Mifepristone in BioUltra 400 polyethylene glycol (PEG) (vehicle) (PEG) was administered subcutaneously (SC) at a dose of 25 mg/kg immediately prior to fasting. As previously described (29), palmitate in 50 mL of castor oil (vehicle) was administered IP at a dose of 30 μmol.

### Quantitative PCR

As we have described (30), animals were perfused with 30 mL of ice cold PBS and RNA isolated from homogenized tissues using Qiagen RNeasy Lipid Tissue Mini Kits (Valencia, CA, USA). RNA was reverse transcribed using the Applied Biosystems High-Capacity cDNA Reverse Transcription Kit. The TaqMan Gene Expression primers used were: IL-1α (Mm99999060_m1), IL-1β (Mm99999061_mH), IL-1R1 (Mm00434237_m1), IL-1RA (Mm01337566_m1), and IL-1R2 (Mm00439622_m1). Quantitative PCR (qPCR) was performed on an Applied Biosystems 7900 HT Fast Real-Time PCR System using Applied Biosystems TaqMan Universal PCR Master Mix. To compare gene expression, a parallel amplification of endogenous RPS3 (Mm00656272_m1) was performed. Reactions with no reverse transcription and no template were included as negative controls. Relative quantitative evaluation of target gene to RPS3 was performed by comparing Δ*C*_t_s, where *C*_t_ is the threshold concentration.

### Locomotion

Spontaneous locomotor activity was measured as we have previously described (22, 31). At the times indicated, mice were video recorded in their home cage for 5 min under red light using a using a Sony Night Shot capable video camera (Minato-ku, Tokyo). Distance moved was quantified using Noldus Information Technology EthoVision XT 7 automated tracking software (Leesburg, VA, USA).

### Social behavior

Social exploration of a novel juvenile was measured as we have previously described (31). In brief, a 4-week-old novel, conspecific male juvenile (challenge) mouse enclosed in a 3″ × 3″ × 3″ wire mesh cage was placed in the home cage of the adult (test) mouse for 5 min. Test mouse-initiated exploratory behavior of the challenge mouse enclosure was video recorded under red light using a Sony Night Shot capable video camera. Time spent exploring the challenge mouse [nose within a 2 cm region of interest (ROI) drawn around the caged juvenile] was quantified using Noldus Information Technology EthoVision XT 7 automated tracking software (Leesburg, VA, USA).

### Blood glucose

Mouse tail blood glucose was determined in duplicate using an Abbott Laboratories AlphaTRAK Blood Glucose Monitoring System (North Chicago, IL, USA) by methods we have previously described (31).

### Serum, liver, and adipose IL-1R2 and IL-1RA

Serum and tissue IL-1R2 was measured using a Millipore Milliplex MAP kit (Billerica, MA, USA) following manufactures instructions. Bead-based fluorescent reporter signal was detected on a Luminex 100 System, where the minimum detectable concentration of IL-1R2 is 4.0 pg/mL (Austin, TX, USA). Serum and tissue IL-1RA was measured colorimetrically. Serum (50 μL) was measured by ELISA using the R&D Mouse IL-1RA/IL-1F3 conjugate kit, where the minimum detectable concentration ranges from 4 to 13 pg/mL (Minneapolis, MN, USA). Liver and adipose IL-1R2 and IL-1RA were measured from freeze-fractured tissues by methods we have previously described (28). In brief, PBS perfused liver and perigonadal adipose tissue was immediately frozen by liquid nitrogen in a freeze fracture buffer containing 10% glycerol, 50 mM NaCl, 1 mM EDTA, 50 mM HEPES (pH 7.4) plus a 1:200 dilution of Millipore protease inhibitor cocktail (Billerica, MA, USA). The fractured tissues were homogenized using the TissueLyser II (Qiagen, Valencia, CA, USA) at a rotational frequency of 30/s for 2 min. Lysates were clarified at 16,000 × *g* for 15 min at 4°C and the supernatant protein concentrations determined using the Bio-Rad DC Protein Assay (Hercules, CA, USA). IL-1R2 and IL-1RA were determined in 25 and 50 μL of supernatant, respectively, as in serum. Liver IL-1R2 and IL-1RA was expressed as picogram of IL-1R2 per nanogram of supernatant protein.

### Plasma corticosterone, free-fatty acids (FFAs), and leptin

Mice were sacrificed immediately after the 24 h fast. Corticosterone was measured colorimetrically using the Abcam corticosterone ELISA kit (Cambridge, MA, USA) in 5 μL plasma. Free-fatty acids (FFAs) were measured colorimetrically in 10 μL of plasma using the Randox NEFA Assay (Antrim, Ireland) and a Beckman-Coulter AU680 (Brea, CA, USA) following manufactures instructions. Leptin was measured colorimetrically using the R&D Systems leptin ELISA kit (Minneapolis, MN, USA).

### Statistics

Individual behavioral experiments were performed on separate cohorts of mice. Biochemical/qPCR experiments were performed on cohorts of mice not subjected to behavioral testing. All data are presented as mean ± SEM. Data were analyzed using Sigma Plot 11.2 (Systat Software, Chicago, IL, USA). To test for statistical differences, one-way and two-way ANOVAs were used with or without repeated measurements where relevant. Tukey’s test was used for *post hoc* pair-wise multiple comparison procedures. Pearson product moment correlation was used to compute correlation coefficient. Where indicated, raw data were transformed to attain normality or equal variance. All statistical analysis included testing for time point × treatment interactions. Statistical significance was denoted at *P* < 0.05.

## Results

### Fasting up-regulates IL-1R2 gene expression in adipose tissue and liver

Table 1 demonstrates the gene expression of IL-1α, IL-1β, IL-1R1, IL-1RA, and IL-1R2 in brain regions (hypothalamus, hippocampus, and cortex), adipose tissue, and liver after a 24 h fast. The largest fold changes between the fed and fasted state were seen in the up-regulation of IL-1RA message in liver and in IL-1R2 message in adipose tissue and liver.

**Table 1:** **Impact of fasting on IL-1α, IL-1β, IL-1R1, IL-1RA, and IL-1R2 gene expression in brain, adipose tissue, and liver**.

Tissue	State	Gene				
		IL-1α	IL-1β	IL-1R1	IL-1RA	IL-1R2
Hypothalamus	Fed	1.000 (0.06, 0.06)	1.000 (0.17, 0.15)	1.000 (0.10, 0.09)	1.000 (0.33, 0.25)	1.000 (0.13, 0.12)
	Fasted	0.567* (0.03, 0.03)	0.487* (0.05, 0.05)	1.484* (0.12, 0.11)	0.920 (0.25, 0.20)	1.104 (0.05, 0.05)
Hippocampus	Fed	1.000 (0.05, 0.05)	1.000 (0.09, 0.08)	1.000 (0.20, 0.17)	1.000 (0.11, 0.10)	1.000 (0.10, 0.09)
	Fasted	0.565* (0.03, 0.03)	0.791 (0.06, 0.05)	1.600* (0.18, 0.10)	1.600* (0.33, 0.27)	0.880 (0.09, 0.08)
Cortex	Fed	1.000 (0.11, 0.10)	1.000 (0.12, 0.11)	1.000 (0.03, 0.03)	1.000 (0.14, 0.13)	1.000 (0.04, 0.04)
	Fasted	0.496* (0.03, 0.02)	0.9018 (0.11, 0.010)	1.319* (0.06, 0.05)	1.066 (0.12, 0.10)	1.061 (0.06, 0.06)
Adipose	Fed	1.000 (0.58, 0.37)	1.000 (0.36, 0.27)	1.000 (0.11, 0.10)	1.000 (0.34, 0.25)	1.000 (0.25, 0.02)
	Fasted	0.295 (0.05, 0.04)	0.167* (0.04, 0.03)	2.334* (0.36, 0.31)	0.774 (0.16, 0.13)	83.424* (79.04, 40.59)
Liver	Fed	1.000 (0.14, 0.12)	1.000 (0.13, 0.11)	1.000 (0.13, 0.11)	1.000 (1.56, 0.64)	1.000 (0.15, 0.13)
	Fasted	0.857 (0.03, 0.03)	0.979 (0.34, 0.25)	3.130* (0.95, 0.70)	68.482* (38.6, 24.7)	9.467* (19.35, 6.36)

Results are expressed as relative fold change in mRNA (ΔmRNA), means (SEM upper, SEM lower); *n* = 4–9. *P < 0.05, significant (one-way ANOVA). Adipose IL-1R2 and liver IL-1R2 transformed to RANK for equal variances.

### Up-regulation of IL-1R2 gene expression in liver and adipose tissue correlates with loss of body weight

**Figure 1:**
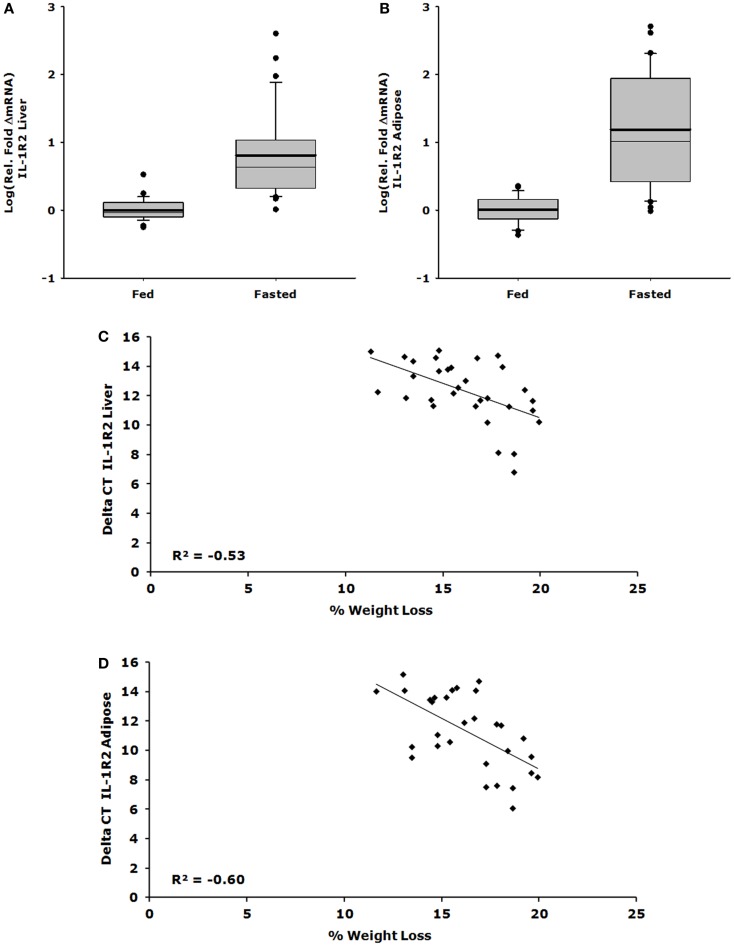
**Up-regulation of IL-1R2 gene expression in liver and adipose tissue correlates with loss of body weight**. Mice were fed or fasted for 24 h. Box plots are representative of the summated relative fold changes in mRNA transformed to log; the box outlines the lower first quartile and upper third quartile, the vertical bars represent the largest/smallest non-outlier observation, the thick horizontal line represents the mean of the relative fold change values, and the thin horizontal line is the median. Black circles represent outliers. **(A)** Liver IL-1R2 gene expression is summarized and expressed as log [average relative fold change (ΔmRNA)] for fed (*n* = 29) and fasted groups (*n* = 31) *P < 0.001. **(B)** Adipose tissue IL-1R2 gene expression is summarized and expressed as log [average relative fold change (ΔmRNA)] for fed (*n* = 29) and fasted groups (*n* = 30) *P < 0.001. **(C)** Percent weight lost per mouse was correlated to delta CT value for IL-1R2 in the liver, significant correlation was observed *P* = 0.002, *n* = 31. **(D)** Percent weight lost per mouse was correlated to delta CT value for IL-1R2 in the adipose tissue, significant correlation was observed *P* < 0.001, *n* = 30.

Figures 1A,C show that IL-1R2 gene expression was up-regulated 26-fold in liver after a 24 h fast (ΔCT fed vs. fasted, 1.082 ± 0.095 vs. 27.975 ± 13.853, *P* < 0.001) and that the correlation between % weight-loss and gene expression was −0.530 (*P* = 0.002). Figures 1B,D demonstrate that IL-1R2 gene expression was up-regulated 62-fold in adipose tissue after a 24 h fast (ΔCT fed vs. fasted, 1.118 ± 0.089 vs. 69.577 ± 21.829, *P* < 0.001) and that the correlation between % weight-loss and gene expression of IL-1R2 was −0.601 (*P* < 0.001). Although IL-1RA gene expression was up-regulated in liver by 28-fold (ΔCT fed vs. fasted, 1.219 ± 0.140 vs. 33.792 ± 6.292, *P* < 0.001) there was no % weight-loss correlation [*R*^2^ = −0.306 (*P* = 0.094)] (data not shown). On average, fasting induced a 12.6% loss in body weight (fed 23.63 ± 0.46 g vs. fasted 20.98 ± 0.46 g *P* < 0.001).

### Fasted mice are resistant to IL-1β

**Figure 2:**
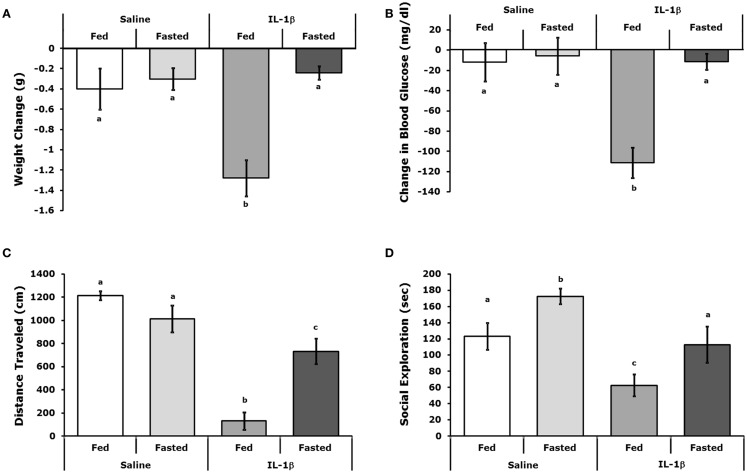
**Fasted mice are resistant to IL-1β**. Mice were fed or fasted for 24 h. prior to IP IL-1β or vehicle (Saline) administration. **(A)** Mice were weighed 2 h after IL-1β administration and compared to their weight just prior to IL-1β administration. Results are expressed as weight change in grams (g), means (±SEM); *n* = 4–5. Bars without a common superscript are different (*P < 0.05). **(B)** Blood glucose was measured 2 h after IL-1β administration and compared to blood glucose just prior to IL-1β administration. Results are expressed as change in blood glucose (microgram/deciliter), means (±SEM); *n* = 4–5. Bars without a common superscript are different (*P* < 0.05). **(C)** Spontaneous locomotor activity was measure 2 h after IL-1β administration. Results are expressed as total distance traveled in centimeters (cm), means (±SEM); *n* = 4. Bars without a common superscript are different (*P < 0.05). **(D)** Social exploration was measured 2 h after IL-1 administration. Results are expressed as total time of exploration (sec), means (±SEM); *n* = 8. Bars without a common superscript are different (*P* < 0.05).

Figures 2A,B show that fasted mice are resistant to IL-1β-induced weight-loss and hypoglycemia [(fed saline −0.400 ± 0.204 g vs. fasted saline −0.300 ± 0.108 g vs. fed IL-1β −1.280 ± 0.180 g vs. fasted IL-1β −0.240 ± 0.067 g); main effects of treatment (*P* = 0.016) and state (*P* = 0.002), treatment–state interaction (*P* < 0.007) and (fed saline −12.000 ± 18.898 mg/dL vs. fasted saline −5.750 ± 18.368 mg/dL vs. fed IL-1β −111.200 ± 14.900 mg/dL vs. fasted IL-1β −11.400 ± 7.619 mg/dL); main effects of treatment (*P* = 0.004) and state (*P* = 0.003) and treatment–state interaction (*P* < 0.007)], respectively. Figures 2C,D demonstrate that fasted mice are resistant to the IL-1β-induced sickness behaviors of reduced locomotion and social withdrawal (fed saline 1213.374 ± 36.764 cm vs. fasted saline 1012.946 ± 115.244 cm vs. fed IL-1β 131.176 ± 74.121 cm vs. fasted IL-1β 731.617 ± 110.055 cm); main effects of treatment (*P* < 0.001) and state (*P* = 0.046), treatment–state interaction (*P* < 0.001), and fed saline 123.5 ± 16.5 s vs. fasted saline 172.4 ± 9.7 s vs. fed IL-1β 62.7 ± 13.1 s vs. fasted IL-1β 112.9 ± 22.3 s; main effects of treatment (*P* < 0.001) and state (*P* = 0.004), no treatment–state interaction (*P* = 0.980)]. Table 2 shows the impact of a 24 h fast on IL-1β-induced changes in gene expression of IL-1α, IL-1β, IL-1R1, IL-1RA, and IL-1R2 in brain regions (hypothalamus, hippocampus, and cortex), adipose tissue, and liver. The largest IL-1β-dependent fold changes seen were in the up-regulation of IL-1RA and IL-1R2 message in adipose tissue and liver. In adipose tissue, fasting suppressed IL-1β-dependent expression of IL-1R1 and IL-1RA message but enhanced expression of IL-1R2 message. In liver, fasting increased IL-1β-dependent expression of IL-1R2 message.

**Table 2:** **Impact of fasting on IL-1α, IL-1β, IL-1R1, IL-1RA, and IL-1R2 gene expression in brain, adipose tissue, and liver of mice treated with IL-1β**.

Tissue	Rx	State	Gene				
			IL-1α	IL-1β	IL-1R1	IL-1RA	IL-1R2
Hypothalamus	Saline	Fed	1.00^a^ (0.18,0.15)	1.00^a^ (0.41, 0.29)	1.00^a,b^ (0.21, 0.17)	1.00^a^ (0.44, 0.30)	1.00^a^ (0.12, 0.11)
		Fasted	0.71^b^ (0.04,0.03)	0.42^b^ (0.07, 0.06)	0.75^a^ (0.04, 0.04)	0.46^b^ (0.04, 0.03)	1.27^a^ (0.04, 0.04)
	IL-1β	Fed	13.5^c^ (0.61,0.59)	72.80^c^ (14.9, 12.4)	1.51^a,b^ (0.30, 0.26)	52.10^c^ (2.89, 2.74)	1.82^b^ (0.19, 0.17)
		Fasted	10.4^c^ (1.22,1.08)	69.31^c^ (9.66, 8.48)	1.19^b^ (0.09, 0.08)	34.20^d^ (3.06, 2.81)	1.87^b^ (0.23, 0.20)
Hippocampus	Saline	Fed	1.00^a^ (0.06,0.06)	1.00^a^ (0.13, 0.11)	1.00^a^ (0.14, 0.13)	1.00^a^ (0.41, 0.29)	1.00^a^ (0.17, 0.15)
		Fasted	0.74^a^ (0.06,0.05)	0.97^a^ (0.04, 0.04)	1.21^a,b^ (0.05, 0.05)	0.87^a^ (0.26, 0.20)	1.15^a^ (0.10, 0.09)
	IL-1β	Fed	7.64^b^ (0.56,0.52)	11.30^b^ (1.35, 1.21)	1.64^b^ (0.08, 0.08)	49.60^b^ (8.32, 7.12)	1.81^b^ (0.25, 0.22)
		Fasted	7.67^b^ (1.57,1.31)	12.60^b^ (2.13, 1.83)	1.54^a,b^ (0.07, 0.07)	31.40^b^ (5.17, 4.44)	2.29^b^ (0.31, 0.27)
Cortex	Saline	Fed	1.00^a^ (0.07,0.07)	1.00^a^ (0.05, 0.05)	1.00^a^ (0.11, 0.10)	1.00^a^ (0.06, 0.06)	1.00^a^ (0.03, 0.03)
		Fasted	0.58^b^ (0.07,0.06)	0.85^a^ (0.11, 0.09)	1.13^a^ (0.12, 0.08)	1.11^a^ (0.17, 0.15)	1.27^b^ (0.07, 0.07)
	IL-1β	Fed	8.91^c^ (0.96,0.86)	11.10^b^ (1.71, 1.48)	2.16^b^ (0.19, 0.18)	23.60^b^ (3.14, 2.76)	1.58^c^ (0.12, 0.11)
		Fasted	7.35^c^ (1.36,1.15)	10.40^b^ (1.48, 1.29)	1.94^b^ (0.16, 0.15)	16.50^b^ (2.57, 2.23)	1.90^d^ (0.10, 0.09)
Adipose	Saline	Fed	1.00^a^ (0.16,0.14)	1.00^a^ (0.39, 0.28)	1.00^a^ (0.24, 0.19)	1.00^a^ (0.25, 0.20)	1.00^a^ (0.38, 0.28)
		Fasted	1.16^a^ (0.83,0.48)	0.84^a^ (0.53, 0.32)	0.68^a^ (0.17, 0.14)	0.62^a^ (0.21, 0.16)	12.9^b^ (8.59, 5.16)
	IL-1β	Fed	9.83^b^ (3.31,2.47)	27.50^b^ (7.11, 5.65)	10.80^b^ (0.63, 0.59)	22.70^b^ (2.70, 2.41)	69.2^c^ (11.0, 9.51)
		Fasted	4.14^b^ (2.38,1.51)	24.60^b^ (6.31, 5.02)	2.97^c^ (0.47, 0.40)	12.35^c^ (1.58, 1.40)	177.9^d^ (39.4, 32.2)
Liver	Saline	Fed	1.00^a^ (0.10,0.09)	1.00^a^ (0.19, 0.16)	1.00^a^ (0.06, 0.06)	1.00^a^ (0.21, 0.17)	1.00^a^ (0.19, 0.16)
		Fasted	0.98^a^ (0.06,0.06)	1.19^a^ (0.28, 0.23)	1.23^a^ (0.38, 0.29)	9.60^b^ (1.00, 0.91)	4.22^b^ (2.31, 1.49)
	IL-1β	Fed	1.24^a^ (0.19,0.17)	4.48^b^ (0.62, 0.54)	7.16^b^ (0.44, 0.41)	463.0^c^ (23.7, 22.5)	124.5^c^ (5.61, 5.37)
		Fasted	1.13^a^ (0.15,0.14)	4.06^b^ (0.84, 0.70)	9.96^b^ (2.23, 1.82)	450.5^c^ (54.5, 48.6)	200.9^d^ (19.0, 17.4)

*Results are expressed as relative fold change in mRNA (ΔmRNA), means (SEM upper, SEM lower); *n* = 8*.

Values without a common superscript are different (*P < 0.05) (two-way ANOVA). Hypothalamus IL-1RA transformed to RANK for normality. Liver IL-1R2 transformed to RANK for equal variance.

### IL-1RA, IL-1R2, FFAs, and corticosterone are increased in fasted mice

**Figure 3:**
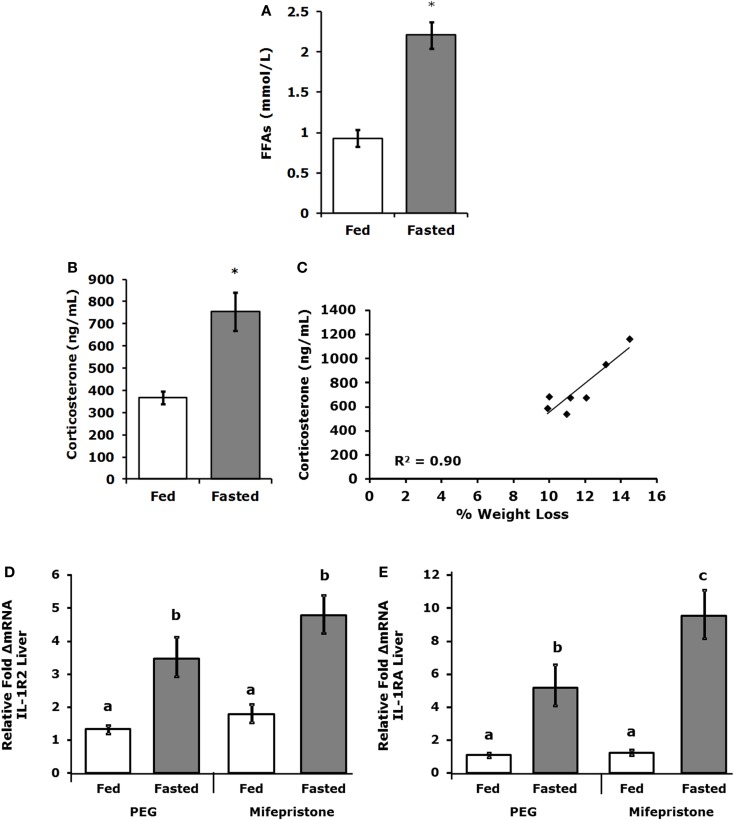
**Free-fatty acids and corticosterone are increased in fasted mice**. Mice were fed or fasted for 24 h. **(A)** Plasma FFAs were measured at 24 h and results are expressed as millimoles per liter, fed vs. fasted, means (±SEM); *n* = 3–4. Significant main effect (*P < 0.001). **(B)** Plasma corticosterone was measured at 24 h. Results are expressed as nanogram per milliliter corticosterone, means (±SEM); *n* = 5–7. Significant main effect (*P < 0.05). **(C)** Plasma corticosterone was correlated to percent weight lost by mouse; *n* = 5–7, *P* = 0.005. **(D,E)** Mice were treated SQ with or without mifepristone then fed or fasted for 24 h. Gene expression of IL-1R2 and IL-1RA were measured in the liver. IL-1R2 gene expression was expressed as relative fold change in mRNA, means (+SEM upper, −SEM lower); *n* = 4–6. Bars without a common superscript are different (*P < 0.05). IL-1RA gene expression was expressed as relative fold change in mRNA, means (+SEM upper, −SEM lower); *n* = 4–6. Bars without a common superscript are different (*P < 0.05).

Table 3 demonstrates that IL-1RA and IL-1R2 are increased by fasting 2.47- and 3.2-fold, respectively, in liver. Fasting increased IL-1R2 in fat 1.78-fold. Fasting did not impact IL-1RA or IL-1R2 in serum. Figure 3A shows that fasting more than doubled plasma FFAs [fed 0.927 ± 0.104 mmol/L vs. fasted 2.207 ± 0.161 mmol/L; main effect of state (*P* < 0.001)]. In turn, fasting decreased serum leptin [fed 15,260 ± 1496 pg/mL vs. 9311 ± 1088 pg/mL (*P* = 0.006)] (data not shown). Fasting also increased plasma corticosterone (Figure 3B) by 2.05-fold [fed 368 ± 29 ng/mL vs. fasted 755 ± 84 ng/mL; main effect of state (*P* = 0.004)]. Figure 3C shows that plasma corticosterone correlated positively with percent mouse body weight lost during fasting (*R*^2^ = 0.9; *P* = 0.005). Figures 3D,E demonstrate that inhibiting glucocorticoid signaling with the glucocorticoid receptor antagonist mifepristone did not prevent up-regulation of IL-1R2 or IL-1RA gene transcripts in the liver after fasting [IL-1R2-fed PEG 1.327, +0.126 −0.115 vs. fasted PEG 3.471, +0.661 −0.555 vs. fed mifepristone 1.789, +0.299 −0.256 vs. fasted mifepristone 4.790, +0.609 −0.540; main effects of treatment (*P* = 0.058) and state (*P* < 0.001) no significant treatment–state interaction (*P* = 0.160): IL-1RA-fed PEG 1.086, +0.152 −0.133 vs. fasted PEG 5.182, +1.395 −1.099 vs. fed mifepristone 1.236, +0.157 −0.140 vs. fasted mifepristone 9.521, +1.587 −1.260; main effects of treatment (*P* = 0.077) and state (*P* < 0.001), no significant treatment–state interaction (*P* = 0.161)]. Blood glucose was used to confirm the effectiveness of mifepristone (fasted PEG 141 mg/dL vs. fasted mifepristone 91 mg/dL).

**Table 3:** **Effect of fasting on IL-1R2 and IL-1RA protein levels**.

Tissue	IL-1R2		IL-1RA	
	Fed	Fasted	Fed	Fasted
Liver	8,475 ± 1,837	27,140* ± 8,993	29,054 ± 3,580	71,722* ± 11,176
Adipose	41,375 ± 4,681	73,788* ± 8,716	630 ± 157	442 ± 277
Serum	11,112 ± 393	10,647 ± 350	662 ± 113	694 ± 96

Results are expressed as (liver picogram IL-1R2 or IL-1RA/nanogram total protein, fat picogram IL-1R2 or IL-1RA/nanogram total and serum picogram IL-1R2 or IL-1RA/milliliter) means (±SEM); liver IL-1R2 *n* = 7–8; liver IL-1RA *n* = 13–16; adipose IL-1R2 *n* = 8; adipose IL-1RA *n* = 8; serum IL-1R2 *n* = 8; serum IL-1RA *n* = 8: *P < 0.05, significant (one-way ANOVA). Liver IL-1R2 protein transformed to log(abs) for normality. Liver IL-1RA protein transformed to log(abs) for equal variance.

### Administration of palmitate increases gene expression of IL-1R2 and IL-1RA in the liver

Table 4 shows that 1 and 12 h after palmitate acid administration gene expression of IL-1RA and IL-1R2 was increased by 11- and 14-fold, respectively, in liver. Whole animal KO of TLR-4, IL-1R1, or IL-4, did not prevent fasting-dependent increases in IL-1R2 (liver and adipose tissue) and IL-1RA (liver), data not shown.

**Table 4:** **Effect of FFA treatment on liver gene expression**.

Time	Treatment	Gene	
		IL-1RA	IL-1R2
1 h	Vehicle	1.000^a,b^ (0.47,0.32)	1.000^a^ (0.23,0.19)
	Palmitate	0.993^b^ (0.48,0.32)	1.594^a^ (0.35,0.29)
12 h	Vehicle	1.702^a^ (1.02,0.64)	2.301^b^ (0.95,0.67)
	Palmitate	10.773^c^ (2.41,1.97)	14.242^c^ (2.06,1.80)

*Results are expressed as relative fold change in mRNA (ΔmRNA) vs. 1 h vehicle treatment, means (SEM upper, SEM lower); *n* = 6. Values with different superscripts are significantly different (*P* < 0.05)*.

## Discussion

Fasting and/or acute starvation causes resistance to LPS-induced sickness behaviors (25, 32, 33) of which IL-1 is a critical effector molecule (34). The importance of IL-1 to LPS-induced sickness symptoms is demonstrated by experiments in which, prophylaxis with IL-1RA prior to LPS administration attenuates sickness behaviors (35). How acute caloric restriction moderates sickness, however, is not clearly understood. Restricting caloric intake is the most effective nutritional intervention for preventing diabesity in rodents, primates, and humans (36), and this intervention appears anti-inflammatory reducing pro-inflammatory biomarkers in the diabetes (36) and mitigating sickness behaviors caused by microbial-derived pathogens in rodents (32). Here, we show that fasting up-regulates the mRNA and protein concentration of both IL-1R2 and IL-1RA with liver being a key repository of IL-1R2 and IL-1RA and adipose tissue being limited to IL-1R2 expression. Previous work suggests that the anti-inflammatory effect of fasting is, in part, related to leptin (33). While leptin may act as a pro-inflammatory (37), its exact role in inflammation is murky (38). As expected, we see a significant decrease in circulating leptin due to our fast. This finding also supports the lack of an IL-1RA increase in adipose tissue. As we have shown, leptin drives IL-1RA production in mouse adipose tissue (39, 40). This leptin-generated IL-1RA originates from fat-based macrophages (41) and is dependent on the long form of the leptin receptor, leptin receptor-dependent activation of the ERK/MAPK pathway and the binding of nuclear factor-κB/PU.1 within the IL-1RA promoter region (42). Since blood leptin is decreased, adipose-derived IL-1RA is expected to be decreased or unchanged, as we see. Furthermore, leptin does not appear to directly regulate IL-1R2 gene expression because the IL-1R2 promoter lacks regions impacted by transcription factors driven by leptin (43). Unexpectedly, liver IL-1RA was up-regulated by fasting, but it did not correlate with % change in body weight as did IL-1R2, where a 15% loss of weight appears to be a threshold for marked IL-1R2 up-regulation. Taken together, these findings suggest that during starvation IL-1R2 is a biomarker of pronounced weight loss.

Sickness behaviors are adaptive changes that occur in response to infection (44) and are, thus, a sensitive indicator of inflammation. Dramatic reductions in food intake are commonly associated with localized and systemic illness (45–47), but sickness-associated anorexia is attenuated if food is restricted prior to IL-1β administration (48, 49). Acute starvation also reduces the fever response to LPS in rats (25). Here, we show that fasting prior to IL-1β administration not only attenuates weight loss but also protects mice from IL-1β-induced loss of locomotor activity and social exploratory behavior. As with almost all animals, mice socially withdraw when sick and spend less time moving (19, 50). Whether these behaviors are a form of energy conservation or an evolutionarily helpful isolation to protect others form the spread of disease, is not known. In that, pre-sickness energy restriction (fasting) blunts these sickness responses allowing for a reduction in “self-imposed” social isolation, self-preservation, and/or energy needs to fight infection may be paramount over protecting others. Thus, our findings support the concept that acute caloric restriction is anti-inflammatory at least within the IL-1-arm of the immune system. Part of IL-1-driven inflammation is the feed-forward effect of IL-1 on IL-1 expression. Here, we show that brain and adipose tissue are especially sensitive to IL-1β-dependent up-regulation of IL-1α and IL-1β. Liver, in contrast, is less affected by IL-1β with IL-1α showing no up-regulation. Interestingly, fasting alone had little impact on IL-1-driven IL-1α or IL-1β message. In terms of IL-1R1, fasting only impacts its gene expression in adipose tissue. Fasting, however, dramatically influences IL-1-driven changes in endogenous antagonist expression. Not only were large fold changes in IL-1RA and IL-1R2 message seen in brain, liver, and adipose tissue due to exogenous IL-1β administration, but under these conditions fasting further augmented IL-1R2 expression in cortex, adipose tissue, and liver. Unexpectedly, fasting blunts IL-1RA gene expression in hypothalamus and adipose tissue. However, because of the importance of IL-1RA to inhibit both IL-1α and IL-1β, some argue that IL-1RA is a biomarker of inflammation severity (51). Given our results, we favor IL-1RA as a somewhat non-specific anti-inflammatory that is critical to dampening runaway inflammation best exampled by cytokine storm seen in some cases of very severe influenza.

Since fasting does not increase serum IL-1RA or IL-1R2, a key question is how might CR-dependent up-regulation of endogenous IL-1 antagonists in tissues create IL-1 resistance. Unlike IL-1RA, which exists in a single form, IL-1R2 exists in two forms one which is membrane bound and one which is soluble (sIL-1R2) (43). As with IL-1RA, IL-1R2 serves as a negative regulator of IL-1 signaling, but instead of blocking the IL-1R1 receptor as does IL-1RA, IL-1R2 competes with the IL-1R1 for IL-1. In addition, IL-1R2 can complex with IL-1 receptor accessory protein (IL-1RAP) to ineffectualize IL-1R1 signaling (43). Intracellular sIL-1R2 has long been known to sequester pro-IL-1β, where it interferes with caspase-1-dependent IL-1β maturation (52). More recently, sIL-1R2 has been shown to complex with the pro-IL-1α inhibiting its enzymatic maturation by calpain (53). Therefore, increased intracellular expression of IL-1R2 blocks maturation of both IL-1α and IL-1β. Such a mechanism would be very important to regulate sickness behaviors because the communication of cytokine-mediated sickness signals from the periphery to the brain is reliant, in part, on *de novo* synthesis of IL-1 (54). This is evidenced in rodents, which have undergone sub-diaphragmatic vagotomy having reduced sickness behaviors and IL-1β mRNA in the brain after IP IL-1β administration (55, 56). Recently, the liver–brain axis has gained importance due to the debilitating sickness systems associated with liver inflammation (54). In that, IF has emerged as a tool in thwarting overnutrition-dependent inflammation (57), increased intracellular sIL-1R2 may be a mechanism by which fasting dampens both liver- and adipose tissue-based inflammation while inhibiting the ability of these tissues to communicate with vagal afferents through mature/secreted IL-1.

Fasting, via activation of the sympatho-adrenomedullary system, generates a metabolic profile that favors utilization of long-chain fatty acids (58). Since glucocorticoids are one of the best described inducers of IL-1R2 expression (43), we used the steroid receptor blocker mifepristone (59) to dampen fasting-induced IL-1R2 and IL-1RA expression. While mifepristone prevented fasting-dependent hyperglycemia (data not shown), it did not reduce gene expression of IL-1R2 or IL-1RA. Due to recent work showing that FFAs can induce sickness-like symptoms in a TLR4-, IL-1R1-, and MyD88-independent fashion (29), we administered palmitate to determine if IL-1R2 and IL-1RA gene expression in liver is induced. The effect was small 1 h after palmitate administration but robust after 12 h. Since fasting increases IL-1R2 and IL-1RA gene expression in TLR4 KO mice, FFAs stimulating TLRs is not a likely mechanism by which fasting augments anti-inflammation. In addition, other physiological processes associated with FFA-induced TLR activation (60) are not described in acute fasting such as induction of TNF-α (61). Thus, FFAs such as palmitate may act as anti-inflammatories via interactions with G-protein receptor (GPR)40 also known as FFA receptor 1 (FFAR1) (62). Such a possibility has been shown *in vitro* with docosahexaenoic acid (DHA) in LPS-treated macrophages, where pretreatment with DHA inhibits caspase-1 activation and IL-1β secretion via a mechanism reliant on GPR120 and GPR40 (63). Taken together, our findings indicate that increases in long-chain fatty acids during fasting may be partially responsible for the immunosuppression-like phenotype observed during fasting/short term starvation by inhibiting the IL-1-arm of the immune system. More work is needed to determine precisely how weight loss triggers up-regulation of endogenous IL-1 antagonist.

## Conflict of Interest Statement

The authors declare that the research was conducted in the absence of any commercial or financial relationships that could be construed as a potential conflict of interest.

## Abbreviations

BSA, bovine serum albumin; CR, calorie restriction; DHA, docosahexaenoic acid; FFAs, free-fatty acids; IF, intermittent fasting; IP, intraperitoneal; IL-1R2, IL-1 receptor type 2; IL-1RA, IL-1 receptor antagonist; IL-1RAcP, IL-1 receptor accessory protein; KO, knockout; LPS, lipopolysaccharide; PBS, phosphate buffered saline; qPCR, quantitative polymerase chain reaction; RT, room temperature; SC, subcutaneous; WT, wild type.
